# Using point-of-care HbA1c to facilitate the identification of diabetes and abnormal glucose regulation in primary healthcare settings

**DOI:** 10.3389/fpubh.2023.1078361

**Published:** 2023-05-09

**Authors:** Linhua Pi, Ying Zheng, Xiajie Shi, Zhen Wang, Zhiguang Zhou

**Affiliations:** ^1^National Clinical Research Center for Metabolic Diseases, Key Laboratory of Diabetes Immunology (Central South University), Ministry of Education and Department of Metabolism and Endocrinology, The Second Xiangya Hospital of Central South University, Changsha, Hunan, China; ^2^Center for Medical Research, The Second Xiangya Hospital, Central South University, Changsha, China

**Keywords:** point-of-care, HbA1c, diabetes, abnormal glucose regulation, primary healthcare settings, China

## Abstract

**Background:**

Glycated hemoglobin A1c (HbA1c) is a critical index for the diagnosis and glycemic control evaluation of diabetes. However, a standardized method for HbA1c measurement is unaffordable and unavailable among the Chinese population in low-resource rural settings. Point-of-care (POC) HbA1c testing is convenient and inexpensive, but its performance remains to be elucidated.

**Objective:**

To investigate the value of POC HbA1c for identifying diabetes and abnormal glucose regulation (AGR) in the resource-limited Chinese population.

**Methods:**

Participants were recruited from 6 Township Health Centers in Hunan Province. Samples for POC HbA1c, venous HbA1c, fasting plasma glucose, and 2 h-plasma glucose were obtained after physical examination. The oral glucose tolerance test was performed as the gold standard for diagnosis. The diagnostic capacities of the POC HbA1c measurement in predicting undiagnosed diabetes and AGR were evaluated.

**Results:**

Among 388 participants, 274 (70.6%) normoglycemic controls, 63 (16.2%) prediabetes patients, and 51 (13.1%) diabetes patients were identified with oral glucose tolerance test (OGTT). Meanwhile, among 97 participants who underwent two HbA1c detection methods simultaneously, a positive correlation was found between POC HbA1c and standardized HbA1c (*r* = 0.75, *P* < 0.001). No notable systematic difference was observed from the Bland-Altman Plots. The POC HbA1c cutoff values were 5.95 and 5.25%, which efficiently identified diabetes (AUC 0.92) and AGR (AUC 0.89), respectively.

**Conclusions:**

The alternative POC HbA1c test efficiently discriminated AGR and diabetes from normoglycemia, especially among the Chinese population in primary healthcare settings.

## Introduction

Diabetes has been a major public health crisis around the world, with more than half a billion people living with diabetes (1). The prevalence of diabetes is high and increasing in China. A national survey indicated that the prevalence of diabetes and prediabetes was 12.8 and 35.2%, respectively, among adults living in China. However, diabetes and prediabetes remain undiagnosed in up to 50% of people with these disorders (1). Early identification of diabetes and prediabetes provides an opportunity to commence effective preventive treatment that leads to improved health outcomes (2–4). Therefore, early identification of diabetes through a reliable and convenient screening test is becoming a major health priority.

Although many scientific societies recommend screening for diabetes in the general population, there is currently no international consensus for screening strategies (5, 6). Three approaches are commonly used to detect diabetes and prediabetes: fasting plasma glucose (FPG), the oral glucose tolerance test (OGTT) and glycated hemoglobin A1c. These three methods have their own advantages and limitations. FPG has been proven to be feasible, convenient and reproducible but has low sensitivity and high preanalytical variability. While the OGTT is verified to be more sensitive, this approach is poorly accepted due to its poor reproducibility, cumbersome procedure and questionable cost-effectiveness (7). The HbA1c test has several advantages, including greater convenience, greater reproducibility and greater stability during illness or stress (7, 8). However, high cost and limited availability in certain regions of developing countries are barriers to using this method widely (9, 10).

Point-of-care (POC) HbA1c can provide rapid “on-site” results using handheld devices and blood samples obtained by fingerstick. The device requires little expertise and is easy to operate with no major procedural challenges. Some studies have proven that it can be used to facilitate the identification of prediabetes and diabetes, especially in resource-limited settings, at a relatively lower cost (11–13). To the best of our knowledge, no relevant studies have been conducted in China. The aim of this study is to quantify the performance of POC HbA1c in identifying undiagnosed diabetes and abnormal glucose regulation (AGR) in an asymptomatic, resource-limited Chinese population.

## Methods

Local primary care providers conducted the study and collected data from January 1 to December 31, 2021, at 6 Township Health Centers in Pingjiang County, Hunan Province. Native residents at least 18 years of age without previously diagnosed diabetes or prediabetes were eligible for the study. Individuals with severe anemia or those who recently experienced massive blood loss were excluded from the study. Native residents without previously diagnosed diabetes or prediabetes who were due for diabetes screening were invited to take part. The cross-sectional study was conducted on a real-world basis according to available resources. All participants provided written informed consent, and the study was approved by the ethics committee of the Second Xiangya Hospital of Central South University.

The sample size required was determined, using the estimate prevalence of AGR detected by OGTT and HbA1c and a formula for a comparative studies. At a significance level of 95%, power of 80%, estimated occurrence of AGR in the general population being 30% and hypothesized difference in prevalence of AGR between the two tests at 17%, the minimum computed sample size was 238 and adjusted to 388.

### Measurements

Clinical staff determined fasting status and performed physical examinations and laboratory tests. Participants underwent physical measurements of weight, height, waist circumference (WC), body mass index (BMI), and blood pressure (BP). Height and weight were measured using a wall-mounted stadiometer and calibrated scales with participants standing up with no shoes and lightly clothed. Height and weight were measured to the nearest 0.5 cm and 0.1 kg, respectively. BMI was calculated as weight in kilograms divided by the square of height in meters. Waist circumference (WC) was measured in the horizontal plane midway between the 12th rib and iliac crest using flexible tape. WC was recorded to the nearest 0.5 cm. Blood pressure measurements were taken using a calibrated electronic BP device (OMRON) with the participant seated. Before the measurement, the participants were asked to sit silently for 5–10 min.

The laboratory assessment included a capillary POC finger-prick HbA1c measurement, laboratory HbA1c, fasting plasma glucose levels (FPG) and two-hour plasma glucose levels (2-h PG) after carrying out an oral 75 g glucose tolerance test. For POC HbA1c measurement, one blood drop was obtained by fingerstick and placed on a separate applicator. Then, a trained nurse performed the HbA1c measurement with a portable HbA1c testing system (Sinocare, China, measurement range 4.0–15.0%). Whole blood samples were collected in EDTA tubes (HbA1c test) and fluoride/oxalate tubes (glucose test). Venous plasma glucose was measured by the glucose oxidase peroxidase method on an automatic biochemical analyzer (Mindray BS-180 Analyzer) at local Township Health Centers. Venous whole blood samples for HbA1c were stored at 4°C and sent to the laboratory of the Second Xiangya Hospital. HbA1c was measured by high-performance liquid chromatography (Bio-Rad VARIANT II Hemoglobin Analyzer), which is certified by the National Glycohemoglobin Standardization Program (NGSP).

OGTT was defined as the gold standard, and the ADA criteria were used to diagnose diabetes and prediabetes. Diabetes was diagnosed when FPG was ≥7.0 mmol/L or 2-h PG was ≥11.1 mmol/during OGTT. Prediabetes was diagnosed when FPG was 5.6 mmol/L to 6.9 mmol/L or 2-h PG during 75-g OGTT was 7.8 mmol/L to 11.0 mmol/L (6). AGR includes diabetes and prediabetes.

### Statistical analysis

Data were analyzed with GraphPad Prism software version 8 (GraphPad Software, San Diego, CA) and SPSS version 25.0 (IBM Corporation, Chicago, IL). Data are presented as the mean ± SD, quartile or percentage of total. A normality test (Kolmogorov-Smirnov test) was performed before the data analysis. Comparisons between groups were made using One-Way Analysis test or nonparametric test. The agreement between venous plasma and POC HbA1c measurement was assessed with the Pearson correlation coefficient. Systematic differences between the HbA1c values obtained from venous plasma and POC HbA1c measurements were evaluated by Bland-Altman Plots (14). Receiver operating characteristic curves (AUCs) were used to determine the ability of POC HbA1c to identify diabetes and AGR. *p* < 0.05 was considered statistically significant.

## Results

A total of 388 participants were recruited, and POC HbA1c and OGTT were performed in all participants, whereas venous HbA1c was completed in 97 of all participants due to the inconvenience of specimen transportation. Of the 388 participants, 63 (16.2%) had undiagnosed prediabetes, and 51 (13.1%) had undiagnosed diabetes based on OGTT. Table 1 summarizes the characteristics of participants according to the different glucose tolerance categories.

**Table 1:** Characteristics of the entire study group and three glucose tolerance categories according to classification by oral glucose tolerance test results.

	**All**	**NGT**	**Prediabetes**	**Diabetes**	***p* value**
No.	388	274	63	51	
Men (%)	215 (55.4%)	148 (54.0%)	40 (63.5%)	27 (55.4%)	0.367
Age (years)	63.0(55.0–70.0)	64.0 (54.0–70.0)	59.0 (55.0–67.0)	65.0 (59.0–70.0)	0.108
WC (cm)	80.0 (75.0–86.0)	78.0 (74.0–83.0)	84.0 (80.0–90.0)	91.0 (80.0–100.0)	<0.001
BMI (kg/m^2^)	23.0 (21.4–24.9)	22.8 (21.3–24.1)	24.4 (21.9–26.4)	23.4 (21.5–27.2)	0.01
SBP (mmHg)	130.0 (124.0–138.0)	129.0 (123.0–136.0)	135.0 (123.0–141.0)	138.0 (128.0–150.0)	0.003
DBP (mmHg)	79.0(76.0–84.0)	78.0 (76.0–82.0)	80.0 (75.0–86.0)	85.0 (78.0–90.0)	<0.001
FPG (mmol/L)	5.0(4.7–5.4)	4.9 (4.7–5.2)	5.6 (4.6–6.0)	7.3(6.4–8.7)	<0.001
2-h PG (mmol/L)	6.8 (6.5–7.4)	6.7 (6.4–6.8)	8.0 (7.0–9.0)	12.5 (10.2–15.3)	<0.001
POC HbA1c (%)	5.2 (5.0–5.9)	5.0 (4.9–5.2)	5.7(5.4–6.2)	6.7 (6.2–7.6)	<0.001

Data are reported as the mean (SD), number (%). NGT, normal glucose tolerance; WC, wait circumference; BMI, body mass index; SBP, systolic blood pressure; DBP, diastolic blood pressure; FPG, fasting plasma glucose; 2-h PG, two-hour plasma glucose level (2-hPG) after carrying out an oral 75 g glucose tolerance test.

### Agreement between POC capillary and standard venous HbA1c measurement

**Figure 1:**
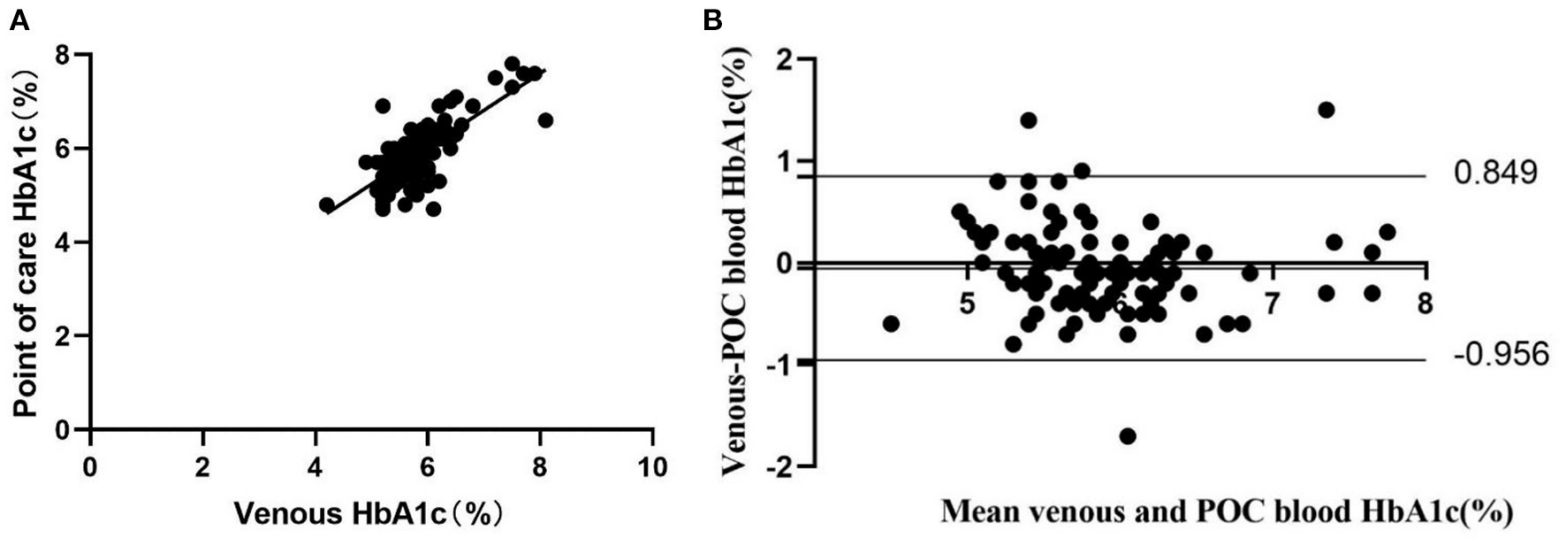
Concordance between laboratory venous blood HbA1c analysis and POC HbA1c analysis (*n* = 97). **(A)** Pearson correlation and scatter plot. **(B)** Difference vs. mean plot (Bland and Altman plot) of capillary blood HbA1c measured by POC and venous laboratory HbA1c measurement. The horizontal top and bottom lines represent ±2 SD.

POC capillary blood and venous HbA1c measurements showed a high positive correlation (*r* = 0.75, *P* <0.001) (Figure 1A). No notable systematic difference was observed from the Bland and Altman Plots at any given blood HbA1c level, only 1 lower outlier and 3 upper outliers outside the agreement limits range (95% confidence intervals: −0.956 to 0.849) (Figure 1B).

### Performance of POC HbA1c to identify diabetes and AGR

**Figure 2:**
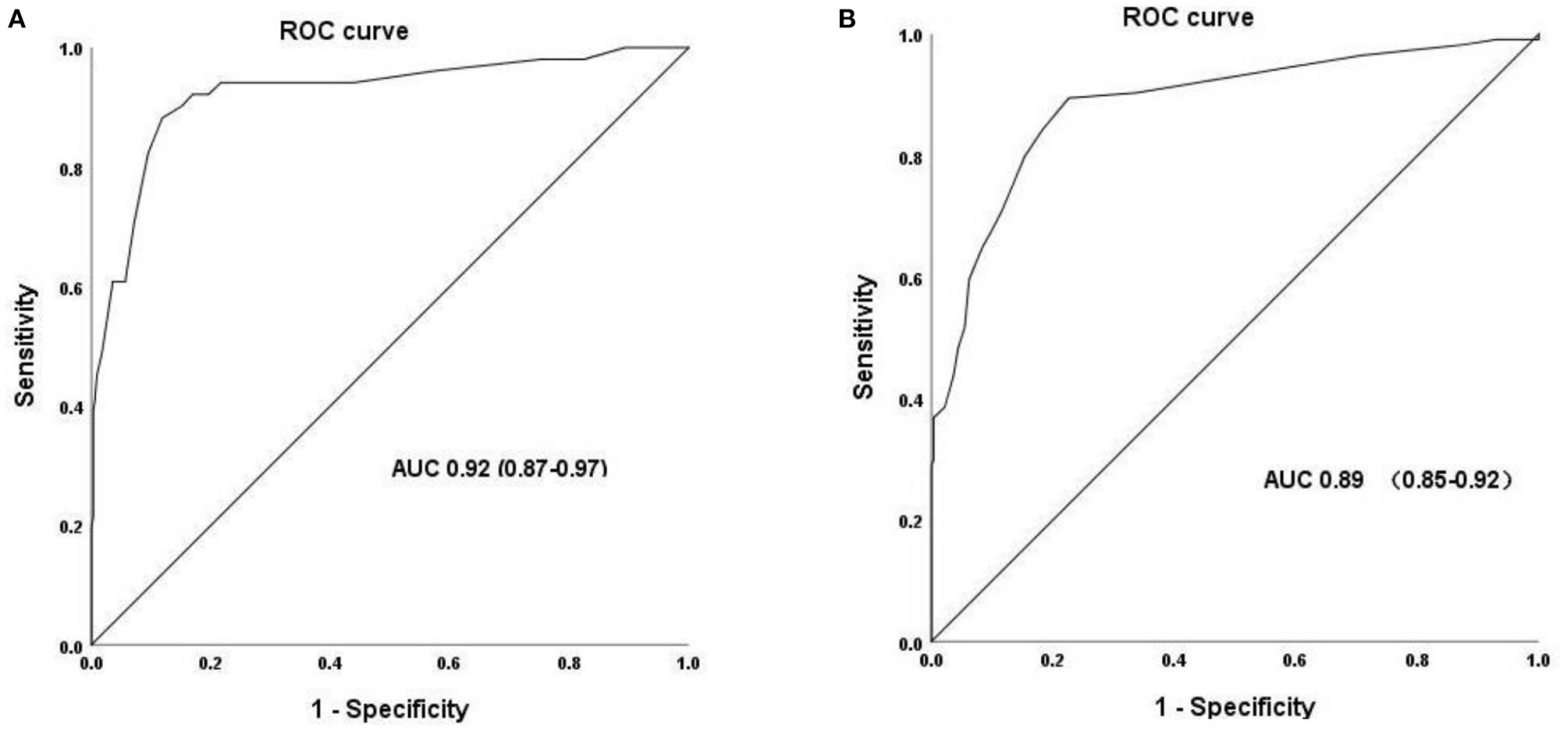
Receiver operating characteristic (ROC) curve for POC HbA1c to identify the presence of undiagnosed diabetes **(A)** and AGR **(B)**.

With the OGTT as the “gold standard” for detection of diabetes and AGR, POC HbA1c tests provided a highly discriminatory capacity for predicting the presence of diabetes with an AUC of 0.92. The most appropriate POC HbA1c threshold value for diagnosing diabetes was 5.95%. The sensitivity and specificity were 88.2 and 88.1%, respectively. The AUC for POC HbA1c tests in predicting AGR was 0.89. The most appropriate POC threshold value for diagnosing diabetes was 5.25% (Figure 2A; Table 2). The sensitivity and specificity were 89.5 and 77.4%, respectively (Figure 2B; Table 2). Meanwhile, the high NPV of POC HbA1c of 94.4% could rule out normal glucose tolerance individuals effectively (Table 2).

**Table 2:** POC HbA1c cutoff points and utility with respect to diabetes and AGR.

	**AUC**	**Cut point (%)**	**Sensitivity (%)**	**Specificity (%)**	**PPV (%)**	**NPV (%)**
Diabetes	0.92	5.95	88.2	88.1	52.3	98.0
AGR	0.89	5.25	89.5	77.4	62.2	94.6

AGR, abnormal glucose regulation; AUC, area under curve; PPV, positive predictive value; NPV, negative predictive value.

### POC HbA1c is less costly and more convenient than the laboratory HbA1c method

The POC HbA1c test is less costly. Furthermore, it only requires one blood drop obtained by fingerstick and can provide rapid “on-site” results within several minutes. In contrast, the laboratory test requires a venous blood sample collected by a trained phlebotomist and 1–2 days to receive a definitive result (Table 3).

**Table 3:** The comparison between HbA1c measurement by POC and the standard laboratory method.

**Item**	**POC HbA1c**	**Laboratory test of HbA1c**
Unit Cost (yuan)^*^	15	45
Time to get the result	5 min	1–2 days
Required blood volume	One drop	2–3 ml
Blood collection method	Finger prick	Phlebotomy

^*^Unit cost in China.

## Discussion

Our study of the utility of POC capillary HbA1c in predicting undiagnosed diabetes and AGR in primary healthcare settings has three findings. First, there was a strong positive correlation between POC capillary HbA1c levels and venous laboratory HbA1c levels. Second, POC HbA1c demonstrated highly discriminatory capacity for identifying undiagnosed diabetes and AGR. Finally, the POC HbA1c test was less costly and more convenient than the venous laboratory HbA1c test.

Currently, the American Diabetes Association (ADA) recommends that diabetes and prediabetes be diagnosed by measuring the fasting plasma glucose (FPG) value or the 2-h plasma glucose (2-h PG) value during a 75-g oral glucose tolerance test (OGTT) or by A1C criteria (6). The HbA1c test has several advantages, including greater convenience, greater reproducibility and greater stability during illness or stress (7, 8). However, these advantages may be offset by the high cost and limited availability in certain regions of developing countries.

POC capillary HbA1c is a more attractive, alternative diagnostic test due to its lower cost and greater convenience. Typically, the POC HbA1c device uses a drop of blood obtained by fingerstick applied to a reagent cartridge, and the analysis is performed in a desktop analyzer. It can provide rapid “on-site” results within several minutes. Expert groups recommended using the POC HbA1c test to monitor glycemic control and guide outpatient decisions for patients with diabetes (6). This testing method can enable more timely treatment adjustments based on the immediate results. It is highly acceptable to physicians and patients. Clinical studies have demonstrated that it can significantly improve glycemic control compared to standard laboratory tests (15–17). However, the utility of POC HbA1c to diagnose diabetes or prediabetes is limited owing to concerns about the accuracy of this method, namely, its accuracy relative to a standard laboratory method (18). No large studies have been performed to evaluate the clinical performance of POC HbA1c testing compared with laboratory HbA1c testing. The reliability of POC HbA1c measurements has been debated since some studies have shown that some POC HbA1c devices may not be suitable for clinical use due to the high variation in accuracy (19, 20). However, some studies have been performed to evaluate the reliability of POC HbA1c devices, and the results showed that POC HbA1c devices, including the Siemens DCA Vantage™, A1C EZ 2.0 (Biohermes, Wuxi, China), etc., met the criteria for accuracy set by the National Glycohemoglobin Standardization Program (NGSP) (21, 22). A similar investigation was also performed in our study. The results showed that POC capillary blood and venous HbA1c measurements showed a high positive correlation (*r* = 0.75, *P* <0.001). No notable systematic difference was observed from the Bland-Altman Plots at any given blood HbA1c level, with only 1 lower outlier and 3 upper outliers outside the agreement limit range. It is recommended that POC HbA1c should not be used to diagnose diabetes unless this method is validated as accurate (6, 23). We verified that POC HbA1c measurements in the present study showed a high level of agreement with laboratory testing and were an alternative method efficiently used to screen and diagnose diabetes or AGR in primary healthcare settings.

Some clinicians have attempted to use POC HbA1c to screen or diagnose diabetes or prediabetes in certain settings or resource-limited regions (11–13). One study in the Australian indigenous population found that using a combination of POC and laboratory HbA1c could simplify diabetes screening in remote areas (11). Another study in a dental setting suggested that POC HbA1c could be a potential tool for abnormal glucose regulation (AGR) screening in a dental setting (12). Furthermore, a study conducted by a clinical pharmacist in America found that POC HbA1c facilitated the identification of prediabetes in a timely and feasible fashion (13). To the best of our knowledge, there is minimal evidence regarding the performance of POC HbA1c testing for screening or diagnosing diabetes or prediabetes in China. With an increasing incidence and prevalence of diabetes, standard laboratory HbA1c instruments are usually inaccessible and unaffordable in the Chinese primary care setting. Accurate and effective POC HbA1c devices are urgently needed. Our data suggest that performing POC HbA1c measurements and using a threshold of 5.95 or 5.25% can provide a high capacity for identifying diabetes (AUC 0.92) or AGR (0.89), respectively. In addition, POC HbA1c using the Sinocare device is less costly and more convenient than standard laboratory testing. This method may be a feasible and reliable method to identify diabetes or prediabetes and contribute to the prevention of diabetes in China, especially in remote, resource-limited rural region primary care settings.

There are several limitations in this study. First of all, this study was performed in one county in China, the results may not be generalized to other parts of China. Secondly, the number of participants were relatively small, further studies with larger participants are required to verify efficacy of POC HbA1c.

## Conclusions

In conclusion, we verified a novel POC HbA1c device (Sinocare) in primary healthcare settings and found that its performance met the criteria for accuracy. In addition, our results support the use of the POC capillary HbA1c test for identifying diabetes or AGR in remote, resource-limited primary healthcare settings. Studies evaluating the cost-effectiveness of introducing POC HbA1c testing are needed prior to generalizing this method.

## Data availability statement

The raw data supporting the conclusions of this article will be made available by the authors, without undue reservation.

## Ethics statement

The studies involving human participants were reviewed and approved by the Ethics Committee of the Second Xiangya Hospital of Central South University. The patients/participants provided their written informed consent to participate in this study.

## Author contributions

XS and ZW designed the study. LP collected the data, conducted the data analysis, and drafted the manuscript. XS, ZW, and ZZ revised the manuscript. YZ carefully edited the revised manuscript. All authors read and approved the submitted version of the manuscript.
